# A Review on Styrene Substitutes in Thermosets and Their Composites

**DOI:** 10.3390/polym11111815

**Published:** 2019-11-05

**Authors:** Yuchao Wu, Mingen Fei, Renhui Qiu, Wendi Liu, Jianhui Qiu

**Affiliations:** 1 College of Transportation and Civil Engineering, Fujian Agriculture and Forestry University, Fuzhou 350108, China; yc121708@163.com (Y.W.); fme1211@foxmail.com (M.F.); 2 Department of Mechanical Engineering, Faculty of Systems Science and Technology, Akita Prefectural University, Akita 015-0055, Japan; qiu@akita-pu.ac.jp

**Keywords:** thermosetting resin, styrene-free, reactive diluent, composites

## Abstract

In recent decades, tremendous interest and technological development have been poured into thermosets and their composites. The thermosets and composites with unsaturated double bonds curing system are especially concerned due to their versatility. To further exploit such resins, reactive diluents (RDs) with unsaturated sites are usually incorporated to improve their processability and mechanical properties. Traditional RD, styrene, is a toxic volatile organic compound and one of the anticipated carcinogens warned by the National Institute of Health, USA. Most efforts have been conducted on reducing the usage of styrene in the production of thermosets and their composites, while very few works have systematically summarized these literatures. Herein, recent developments regarding styrene substitutes in thermosets and their composites are reviewed. Potential styrene alternatives, such as vinyl derivatives of benzene and (methyl)acrylates are discussed in details. Emphasis is focused on the strategies on developing novel RD monomers through grafting unsaturated functional groups on renewable feedstocks such as carbohydrates, lignin, and fatty acids. This review also highlights the development and characteristics of RD monomers and their influence on processability and mechanical performance of the resulting thermosets and composites.

## 1. Introduction

**Figure 1:**
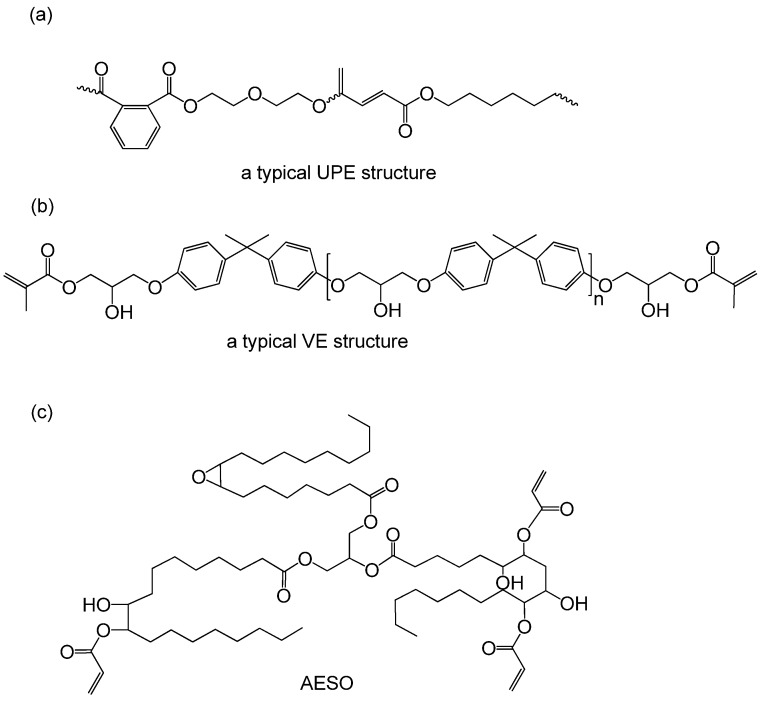
Representative structure of (**a**) unsaturated polyester (UPE), (**b**) VE [3] and (**c**) acrylated epoxidized soybean oil (AESO) [6].

The fast growth of chemical engineering has driven a wider industrial utilization of thermosets cured from unsaturated sites, including unsaturated polyester (UPE), vinyl ester (VE), and triglyceride-based resins. UPE is a linear polymer with unsaturated C=C bonds resulted from saturated di-alcohols with saturated or unsaturated di-acids via a condensation polymerization (Figure 1a) [1]. It is welcomed because of its low cost, high strength, excellent chemical corrosion resistance, good thermal and electrical properties [2]. Bisphenol-A VE is prepared from methacrylic acid and bisphenol-A epoxy resin (Figure 1b) [3,4]. The fast crosslinking process of C=C bonds gives the materials favorable mechanical properties and outstanding chemical and water resistance. In considering the environmental impact and energy consumption, thermosetting resin from vegetable oils is a greener alternative to get rid of using fossil fuels. Its various triglycerides combined with three long-chain fatty acids and glycerol have different length of chains and a range of unsaturation degree, leading to the distinction of curing behavior [5]. As a typical example, acrylated epoxidized soybean oil (AESO) is one of the commercially available vegetable-oil-based resins (Figure 1c) [6]. These commercially available or synthesized resins and their composites are attractive for respective merits and have been widely used in coating, adhesive, automobile parts, turbine blade, etc. [7,8,9,10,11]. 

Upon these resins mentioned above, all of them refer to the free-radical polymerization of unsaturated C=C bonds from main molecular chains. The large or long main chain structure leads to a strong intermolecular force, which is significantly harmful to resin processability and efficiency of crosslink. For example, UPE is solid at room temperature, which makes it hard to process and disperse uniformly for wetting fibers well. The flexible long fatty chains in AESO result in a low mechanical property. Therefore, in common cases, introducing small molecular comonomers as RDs into crosslink network is necessary to enhance their performance at service. 

Currently, styrene is a traditional and preferential RD that has advantages such as low cost, low molecular weight, high reactivity, and excellent polymerizability [12,13,14,15]. With the aid of styrene, improvements occur in resin processability including reduced viscosity and curing temperature as well as increased curing rate and degree, thus leading to better mechanical properties and durability [16]. However, styrene is classified as a volatile organic compound (VOC) for low boiling point and high saturated vapor pressure. Even worse, styrene is not environmental-friendly as a hazardous air pollutant and is harmful to human health [17,18]. The Environmental Protection Agency of USA has legislated to limit styrene emission content in composite fabricating workshop (<50 ppm) [19]. In addition, the National Institute of Health of USA listed it as a potential carcinogen in 2011 and emphasized it again in 2016 [20]. Therefore, it is highly desired to reduce styrene emission or to replace styrene with other greener monomers during the preparation of thermosets and their composites.

In this review, we classify RDs into typically crosslinkable monomers and novel monomers derived from renewable materials. These monomers usually possess a range of characteristics: (1) at least one unsaturated site for participating in crosslinking; (2) good compatibility for making homogeneous resin; (3) low volatility for avoiding the emission of VOC. It can be regarded as low volatile crosslinker in thermosets, and other functions may be bestowed on it as well. The research on greener RDs has practical engineering benefits like improving product performance, satisfying requirements of sustainable industry.

The typical monomers include vinyl derivatives, (methyl)acrylate derivatives, and others. Among vinyl monomers, the most common one is divinyl benzene (DVB), which has a profound application in UV curing coatings. *N*-vinyl-2-pyrrolidone (NVP), *tri*(ethylene glycol)divinyl ether (TDE), and dimethyl itaconate (DI) are also investigated as styrene alternatives. (Methyl)acrylates such as trimethylolpropane triacrylate (TMPTA), trimethylolpropane trimethacrylate (TMPTMA), and methyl methacrylate (MAA) have been investigated for styrene replacements; meanwhile, some RDs such as hydroxyethyl methacrylate (HEMA) and hydroxypropyl acrylate (HPA) have raised more concern. The obtained resins and composites present for responding to the attempts on using typical monomers, which will be further exhibited and compared in this review.

Novel monomers synthesized from renewable materials are extremely attractive. According to its chemical composition, the biobased monomers can be divided into the following categories: (1) oxygen-rich monomers, such as carboxylic acid and furan; (2) hydrocarbon-rich monomers, such as vegetable oil, terpenes, and rosin acid; (3) hydrocarbon monomers, such as biovinyl, biopropylene, bioisopropylene, and biobutylene [21]. They attract wide attention from research and industrial circles for their inherent advantages including low cost, easy availability, renewability, and biodegradability. The Pacific Northwest National Laboratory (PNNL) and the National Renewable Energy Laboratory (NREL) of USA concluded 12 kinds of potential renewable building blocks [22]. Bozell et al. further commented on the top 10 biobased products from biorefinery that were affirmed by the Department of Energy, USA [23]. However, these biobased monomers are unable to perform free-radical polymerization and hence many strategies were developed for introducing unsaturated sites to synthesize crosslinkable monomers. The commonly adopted routes for bestowing them unsaturated groups are classified and discussed detailly in this review. The simple comparison on material performance between products will be given as well.

The purpose of this review, therefore, is to provide an up-to-date overview of the field by highlighting and discussing the latest advances in RDs for styrene-free thermosets and their composites, covering both traditionally crosslinkable monomers and newly developed monomers from renewable feedstocks.

## 2. Traditionally Crosslinkable Monomers

Most of traditionally crosslinkable monomers are industrially available and hence easily utilized as styrene replacements in resins and composites in a large scale. Although these RDs are mostly derived from fossil fuels and toxic as styrene, they still have advantages over styrene such as high boiling point and polymerization efficiency. Their products also show superior properties compared to styrene analogues.

### 2.1. Vinyl Monomers

#### 2.1.1. Divinylbenzene

As a derivative of benzene, divinylbenzene (DVB) is similar to styrene in structure and is a well-studied comonomer that can partially or completely replace styrene since early this century. DVB has two unsaturated double bonds, low molecular weight, and high reactivity (Table 1). However, DVB can easily occur self-polymerization and is not easy to blend with the resins, producing white particles because of too high reactivity. Also, the toxicity of DVB used as crosslinking agent is not entirely clear [24]. 

Zhan et al. [25] investigated the free-radical copolymerization of AESO with styrene and DVB to prepare soybean oil-based thermosets. It was found that DVB could reduce resin viscosity, promote gel process, and increase crosslinking degree. DVB addition resulted in a 14~24 °C increase in the glass transition temperature (*T*_g_) of the resulting resins. Also, the addition of DVB increased the modulus of AESO resin and widened the glass transition zone. The curing kinetics of styrene mixed with DVB as crosslinking agents in AESO resin was further studied [26].

Moreover, DVB has been used in other oil-based resins including fish oil and tung oil [27]. The mechanical properties of the cationically polymerized DVB-based resins were similar to those of styrene-polymerized resins. Larock et al. studied bulk cationic copolymerization of styrene and DVB with conjugated natural oil for the production of agricultural fibers-reinforced composites. The composites were much more brittle, but stiffer than the pure resins. The prepared composite has a renewable content of 88%, and thus has great potential applications in automotive, construction and furniture industries [27].

Other styrene derivatives include α-methylstyrene, fluorostyrene and vinyltoluene (Table 1). Compared to styrene, α-methylstyrene shows a lower reactivity and thus lower crosslinking efficiency of unsaturated double bond in resin system. Fluorostyrene-crosslinked polyesters had better heat resistance and electrical properties. Due to its high boiling point and less volatility, vinyltoluene is an ideal crosslinker, but its price is too high and thus has not been widely used [28].

#### 2.1.2. Other Vinyl Monomers

More recently, two new types of vinyl monomers, i.e., NVP and TDE, were selected to replace styrene in UPE resins for the production of hemp fiber reinforced composites, respectively [29]. As given in Table 1, NVP has a C=C bond that links with nitrogen, giving the C=C bond with a high reactivity due to the electronegativity of nitrogen. TDE has two C=C bonds, which has more opportunities for crosslinking with UPE (Table 1). The results indicated that both monomers could effectively dissolve solid UPE, reduce the entanglement of UPE chains, and significantly reduce the viscosity of the resulting resins (from 50 to 500 mPa·s at room temperature). NVP-based resin composites showed comparable tensile and higher flexural properties with styrene-based resin composites because NVP is a highly reactive monomer. 

Following this work, NVP was further utilized as a RD for AESO resins for preparing the hemp fiber reinforced AESO composites with high performance [30]. It was found that NVP-AESO resins had better processability than styrene-AESO resins, and hemp fiber-reinforced NVP-AESO composites achieved superior static and dynamic mechanical properties when compared to styrene analogues. To improve the crosslinking degree of AESO matrix and its interfacial adhesion with hemp fibers, isophorone diisocyanate (IPDI) was further incorporated into the hemp fibers/AESO composite system [31]. Due to the chemical characteristic of AESO, the IPDI could serve as two functions in the composite system: one is crosslinker within AESO resin for increasing its crosslinking density; the other is coupling agent between hemp fibers and AESO thus giving rise to the interfacial bonding of the composites. The comparison on tensile-fractured surfaces of composites intuitively showed that the addition of diisocyanate improved the interfacial adhesion between fibers and resins. The tensile and flexural properties, storage modulus, and glass transition temperature of the composites were accordingly increased by significantly enhanced interface and crosslinking density.

### 2.2. (Methyl)acrylate Monomers

(Methyl)acrylate monomers are commonly obtained from unrenewable resources. Generally, they have highly reactive (methyl)acrylate C=C bonds and relatively low molecular weight (Table 2).

(Methyl)acrylates have been widely used in the production of coatings, elastomers, adhesives, thickeners, amphoteric surfactants, fibers, plastics, textiles and inks. Butyl methacrylate (BMA) can not only be used as crosslinking agent to reduce styrene content, but also toughen the obtained composites for its soft long molecular chains [2]. The incorporation of BMA into hemp fibers reinforced UPE composite systems significantly increased the toughness of UPE matrices and thus the resulting composites. As a highly polar monomer, hydroxypropylacrylate (HPA) was usually used to control the thermal shrinkage of UPE resin, but its high affinity with organic agent caused insufficient crosslinking, thus negatively affecting the mechanical properties of resins [32]. In a novel family of UPE derived from 2,5-furandicarboxylic acid and itaconic acid, 2-hydroxyethylmethacrylate (HEMA) was selected for copolymerizing with such newly biobased UPE to improve its fluidity and crosslinking degree; results indicated that the increase of HEMA content led to the increases of gel content and *T*_g_ of UPE resins [33,34]. However, HEMA contributed to the increase in swelling capacity of the resins, which was likely due to its high hydrophilicity. It is worth to mention that HEMA could be applied in the preparation of devices used in biomedical applications due to its favorable biocompatibility [35]. In the work of Meht et al. [36], HEMA, isobornyl methacrylate (IBOMA) and methyl methacrylate (MMA) were used as RDs for itaconic acid-based UPE, respectively; results showed that these styrene-free biobased UPE resins had properties equivalent to commercial styrene-based resins. Therefore, these methacrylates could not only replace styrene to reduce resin viscosity, but also improve resin crosslinking density and provide feasible coating properties. However, segments of such monomers may set bond into 3D network and is easy to break down at lower temperature, and thus the HEMA-resulting thermosets are less thermally stable than pure UPE resins [33,34,36]. Also, BMA, HPA and HEMA had low boiling points and thus might easily evaporate during processing. 

Nebioglu et al. [37] investigated the free-radical polymerization kinetics of a multifunctional acrylate monomer, i.e., trimethylolpropanetriacrylate (TMPTA) with UPE. Furthermore, TMPTA was further used as a RD for curable acrylated polyester based hybrid coatings [38]. It was found that the fracture toughness of the composites is the highest when 15% TMPTA was incorporated. With similar chemical structures, the impact of unsaturated sites of RDs on AESO resin has also been discussed by comparing 1,4-butanediol dimethacrylate (BDDMA) and trimethylolpropane trimethacrylate (TMPTMA) in bamboo fibers reinforced AESO composites [39]. The results indicated that the methacrylate RDs with more double bonds endowed the resulting resins with higher crosslinking density. These methacrylates had high molecular weight and thus boiling point, which exhibits almost no VOC emissions during processing. 

### 2.3. Other Monomers

**Figure 2:**
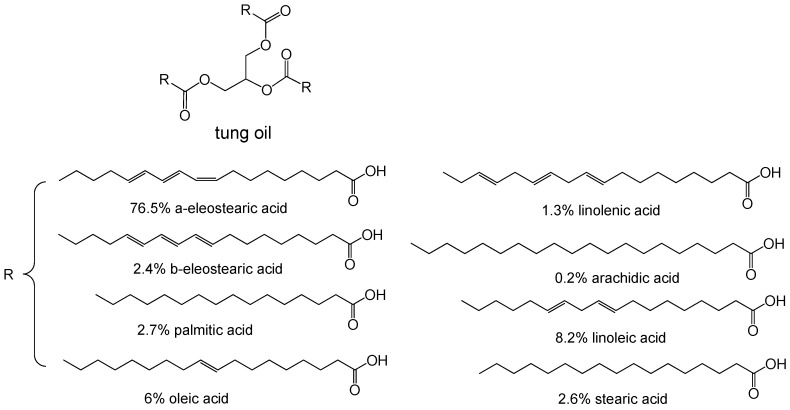
Chemical structure of tung oil.

Tung oil is a drying oil obtained by pressing the seed from the nut of tung tree. Tung oil hardens upon exposure to air and has been mostly used for coatings [40,41]. The fatty acids of tung oil consist of eleostearic, palmitic, oleic, linoleic acids and others (Figure 2). Due to the conjugated double bonds in eleostearic acid, tung oil can directly participate in the crosslinking of some thermosetting resins. It was reported by Meiorin et al. [42] that tung oil was cationically copolymerized with AESO, methyl ester of tung oil, and styrene to produce vegetable oil-based thermosets. The fully tung oil-based resins presented comparable dynamic mechanical properties with styrene analogues. The addition of low concentrations of DVB (5~10 wt%) in the resins increased their rigidity and *T*_g_.

Mistri et al. used 20 wt% tung oil to copolymerize with maleated castor oil (MACO) for fabricating jute fibers reinforced composites [43]. The prepared MACO composites had 42% higher impact strength than that of jute fibers reinforced UPE composites. The flexural modulus of MACO composites was almost similar to that of UPE composites. Besides, the MACO composites exhibited higher damping behavior at room temperature and high temperature. Such composites can be explored further as environmentally friendly and high damping materials over a wide range of temperature [43].

## 3. Novel Monomers Synthesized from Renewable Materials

Renewable materials have been widely investigated to develop novel crosslinkable monomers. As summarized in Table 3, the functionalization methods of renewable monomers are classified as grafting acrylate, grafting methacrylate, and others.

### 3.1. Grafting Acrylate

**Figure 3:**
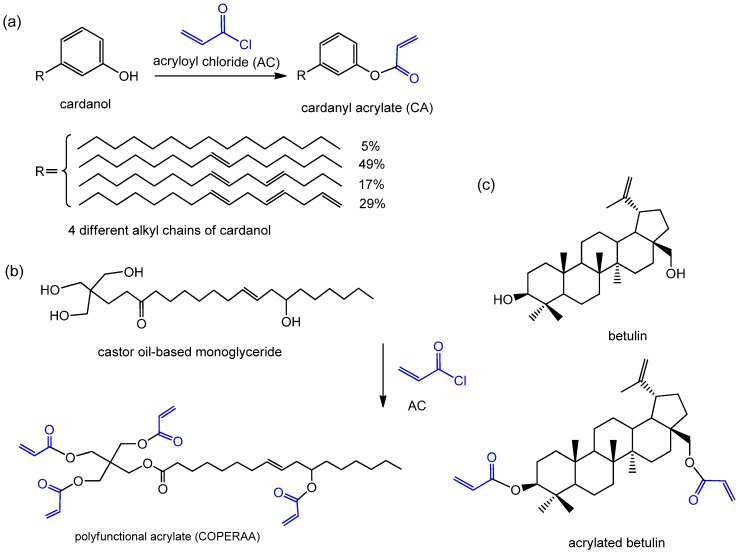
Synthesis routes of (**a**) CA, (**b**) COPERAA and (**c**) acrylated betulin from cardanol, castor oil and betulin with AC [44,45,46,47].

Acryloyl chloride (AC) is a highly reactive monomer and usually used in esterification. The reaction activity of AC with alcohols is much higher than that of anhydride/acid. Cardanol is a phenolic lipid obtained from anacardic acid which is the main component of cashew nutshell liquid, a byproduct of cashew nut processing. Cardanol consists of different saturated and unsaturated long-chain phenols. The unsaturated functionality endows it potentiality to perform free-radical polymerization. The phenolic hydroxyl groups could be used for introducing more C=C bonds. The rigid structure of cardanol would effectively enhance the thermal stability of thermosets. Hu et al. [44] synthesized cardanyl acrylate (CA) from cardanol via reacting with AC for the modification of a castor oil-based polyfunctional polyurethane acrylate resin (Figure 3a). The *T*_g_, thermal stability, hardness, and hydrophobicity of the resins were greatly enhanced by the incorporation of CA. Therefore, CA can replace petroleum-based monomers to produce biobased coatings with special properties such as high biobased content and low shrinkage. Liu et al. [45] successfully synthesized a new polyfunctional acrylate monomer (COPERAA) from castor oil-based monoglyceride and AC (Figure 3b). The COPERAA was used as a RD for AESO UV-curing materials. Compared to petroleum-based TMPTA, COPERAA endowed superior biobased content and volume shrinkage of the resulting AESO resins. AESO/COPERAA resins had lower hardness, thermal stability than AESO/TMPTA resins due to the longer and softer fatty acid chains. Similar designs have been reported by Auclair et al. [46,47]; they selected AC-grafted betulin as a comonomer for preparing AESO coatings with preferable performance (Figure 3c).

### 3.2. Grafting Methacrylate

**Figure 4:**
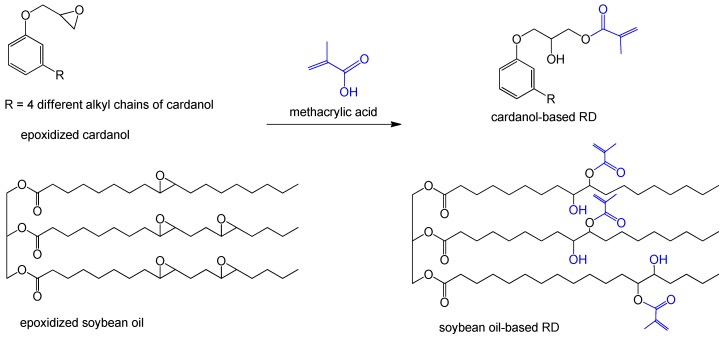
Synthesis of biobased methacrylates from epoxidized cardanol and soybean oil with methacrylic acid [48].

Methacrylic group is another functionality that was used to graft on renewable monomers. Methacrylic acid is one of the simplest approaches for equipping monomers with C=C bonds by esterification. The functionalization of epoxidized cardanol and soybean oil with methacrylic groups were directly conducted with methacrylic acid via an epoxy-acid esterification (Figure 4) [48]. The methacrylic monomers were copolymerized with methacrylated dicyclopentadiene prepolymer; the obtained VE networks presented a *T*_g_ from 100 to 130 °C. Can et al. prepared cardanol-based thermosetting resin by using methacrylated cardanol (MC) as RD for VE resin [49]. The increasing addition of cardanol-based RD from 0 to 50 wt % significantly decreased the viscosity of both commercial and synthesized fully biobased VE resins, but its long chains weakened the crosslink network and mechanical performance.

**Figure 5:**
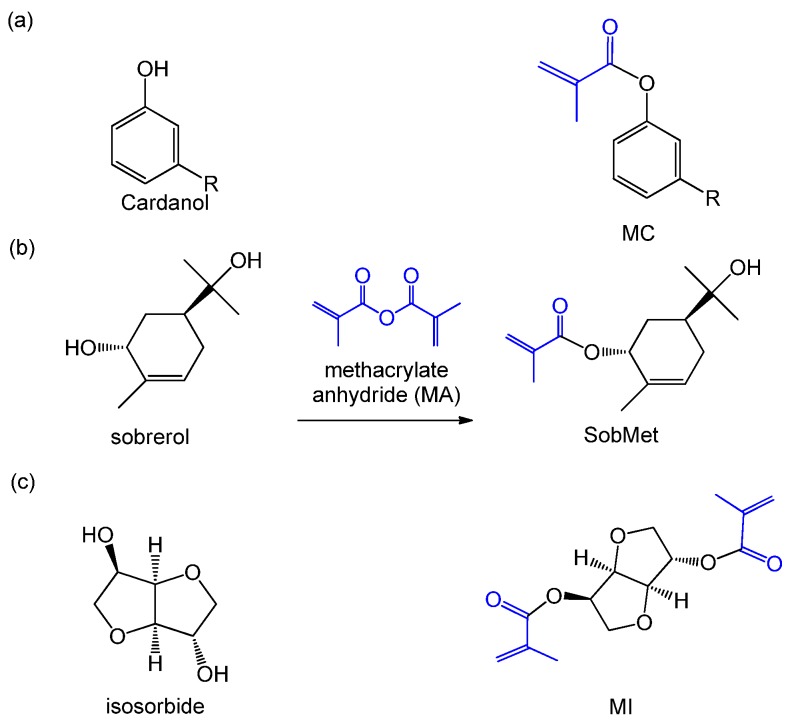
. Synthesis of biobased reactive diluents (RDs) from (**a**) cardanol, (**b**) sobrerol, and (**c**) isosorbide via grafting with MA [50,51,52].

Methacrylate anhydride (MA) is another commonly used monomer for esterification with alcohols. Take a simple example: similar to the route of using methacrylic acid, MA was utilized to graft double bonds onto cardanol for the synthesis of MC as a RD for commercial and synthesized VE (Figure 5a) [50]. Lima et al. [51] synthesized sobrerol methacrylate (SobMet) from sobrerol with MA (Figure 5b), and the new monomer is an interesting alternative for styrene in UPE formulations. SobMet-based resins presented higher *T*_g_ and comparable storage modulus than styrene-containing resins. Hence, SobMet exhibited promising properties to be used as a low-volatile, biobased styrene substitute. Noteworthy, rigid isosorbide could be synthesized through cellulose and starch hydrolysis to glucose followed by hydrogenation to sorbitol and subsequent dehydration to isosorbide. In our previous research, isosorbide was methacrylated and copolymerized with flexible AESO to gain the resins with higher flexural properties, thermal properties, crosslinking density and curing efficiency (Figure 5c) [52]. The achieved resins were further used as matrices for hemp fibers and bamboo fibers to prepare green biocomposites [62]. Results indicated that the resulting composites obtained comparable performance to petroleum-based composites. 

**Figure 6:**
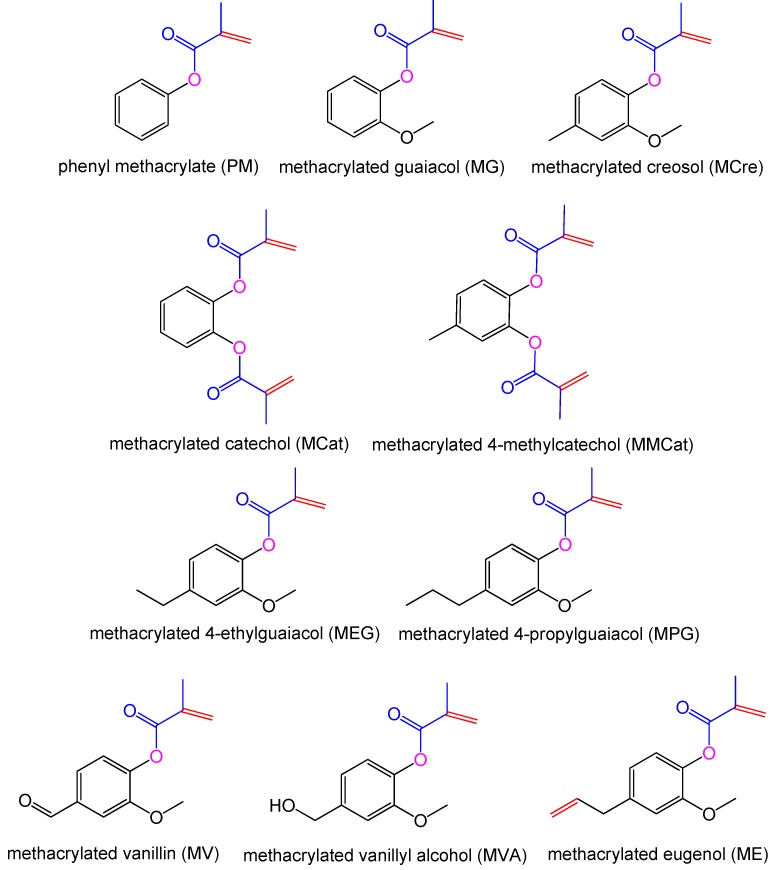
Novel RDs from lignin derivatives [53,54,55,66].

Notably, as novel biobased RDs for thermosets, MA-methacrylated lignin derivatives (Figure 6) became very attractive during past 10 years. This is because lignin is the second most abundant polymer in nature, which is only less than cellulose. New catalytic methods for the efficient production of aromatic monomers from lignin are usually classified as: (1) isolation of lignin by lignocellulosic fractionation prior to catalytic treatment, (2) reductive catalytic fractionation using lignocellulose in the presence of a catalyst, and (3) complete conversion of all lignocellulosic components by a one-pot catalytic process [63]. Especially, under the reductive depolymerization, the yield of the product is greatly increased; many monomers including phenol, guaiacol, eugenol and catechol could be achieved [64]. Wool and his fellows used MA to modify vanillin, guaiacol, and eugenol to generate methacrylated lignin model compounds for VE resins. All the lignin-based resins had relatively high *T*_g_, low viscosity, and low VOC emission, which meets the requirements for liquid molding technique [53]. Furthermore, as a byproduct of esterification between MA and vanillin, methacrylic acid was further used to react with glycerol methacrylate for forming a liquid copolymerized crosslinking agent, i.e., glycerol dimethacrylate [65]. Additionally, a series of methacrylated lignin-based bio-oil mimics were comprised of methacrylated phenol, guaiacols and catechols and utilized as low viscosity VE resins and RDs in typical VE resins [54]. Further work was conducted on the influence of impurities in methacrylated phenolic compounds from lignin [66]. Results suggested that the substitutes on the aromatic ring of the RD have less effect, but the purity of the RD strongly influences the performance of materials. Kessler et al. used vanillin and vanillyl alcohol as feedstocks for reacting with MA. The obtained biobased and low viscosity monomers were blended with VE and maleinated AESO resins, respectively [55,67]. The flexible long chains on AESO would compensate for the brittle drawback on methacrylated vanillin (MV) blended resins. The same method was used to synthesize methacrylated eugenol (ME) as a sustainable RD for maleinated AESO to produce resins with low viscosity, fast curing and superior mechanical performance that were suitable for pultrusion process [68,69]. Dai et al. [70] applied another lignin-based monomer, i.e., methacrylated guaiacol (MG), as a low emission crosslinking agent for curing with novel full biobased UPE resin. Due to the development of additive manufacturing, MG and vanillyl alcohol dimethacrylate are also used to mix with other acrylates for stereolithography 3D printing in recent years [71].

**Figure 7:**
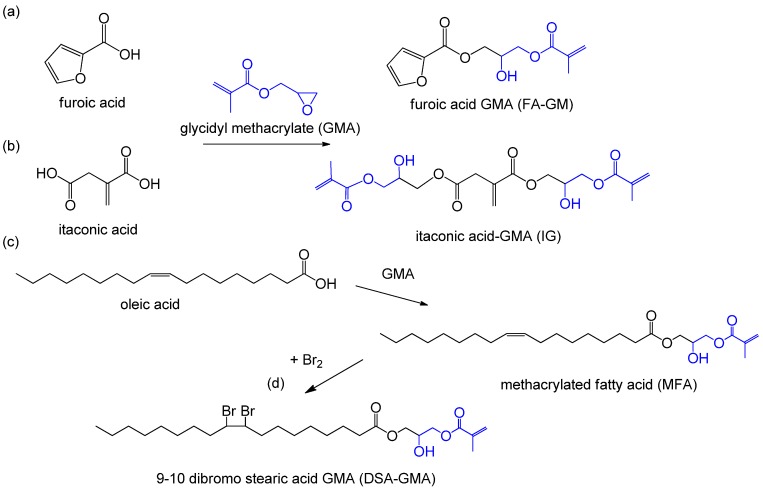
Synthesis of biobased RDs from (**a**) furoic acid, (**b**) itaconic acid, and (**c**,**d**) oleic acid via grafting with GMA [3,56,57].

Glycidyl methacrylate (GMA) is a bifunctional monomer with epoxy group and C=C bond. The epoxy group of GMA gives the opportunity for endowing the target monomers unsaturation functionality through epoxy ring-opening reaction (Figure 7). Furan is a renewable monomer derived from pentose that is obtained from the hydrolysis of corn, oats, and hemicellulose. Both furfuryl alcohol and furoic acid were derived from furan. Furoic acid glycidyl methacrylate (FA-GM) was synthesized from GMA and furoic acid (Figure 7a) and then used as a RD for VE resins. Results indicated that the FA-GM monomer could improve the crosslinking density of resins, leading to admirable curing property with less usage of RD [56]. Dai et al. [57] synthesized a UV-curable unsaturated monomer (IG) from itaconic acid with GMA (Figure 7b). IG could be used together with methacrylates and styrene to significantly improve the strength, modulus and thermal stability of AESO-based resins. Besides, IG was also used as RD for AESO and monomethyl itaconated epoxidized soybean oil for comparison with TMPTMA and styrene [72].

Methacrylated fatty acid (MFA) was obtained by reacting GMA with caprylic acid, caproic acid, oleic acid, linoleic acid and lauric acid from vegetable oil. Oleic acid was selected to present the typical reaction route as shown in Figure 7c. Campanella et al. [73] used methacrylated fatty acid (MFA) as a RD for copolymerizing with maleinated AESO. However, due to the long chain characteristic of fatty acid, the *T*_g_ and storage modulus of the MFA-resulting resins were lower than those of styrene-based resins. Thus, it is still needed to blend with styrene to reduce resin viscosity and balance properties [74]. During the synthesis process of MFA, bromine atoms was grafted to form 9-10 dibromo stearic acid glycidyl methacrylate (DSA-GMA), functionalizing the monomer with improved heating and flame resistance (Figure 7d) [3]. Scala et al. reported that the MFA-based RDs were applied to improve the fluidity of resins, reduce the emission of volatile organic compound and keep the practicability of materials, which were gradually used in military application [75]. Dey et al. also conducted a similar study to prepare MFA by changing the length of basic fatty acid chain [76]. It was found that the viscosity of VE/MFA resin increased with the increase of fatty acid chain length. Hydrogen bonds between MFA hydroxyl groups are affected by the chain length of fatty acids and play a crucial role in controlling the viscosity of the system. However, the *T*_g_ of VE/MFA resin increased with the decrease of fatty acid chain length, which was due to the decrease of free volume of polymer and effective molecular weight between crosslinks.

**Figure 8:**
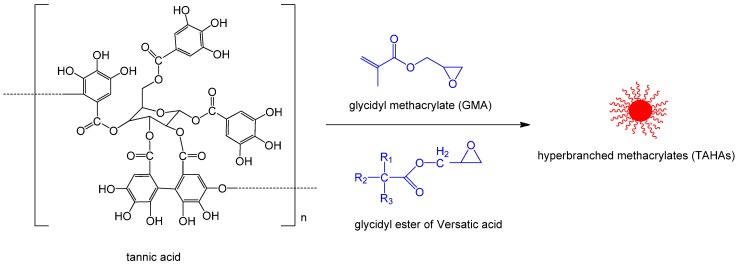
Synthesis of TAHAs from tannic acid via grafting with GMA and CE10 [58].

The monomers containing phenolic hydroxyl groups could also react with the epoxy rings of GMA. Tannic acid is a water-soluble polyphenolic compound with high molecular weight and abundant phenolic hydroxyl groups. The reactivity of phenolic hydroxyl groups is much higher than that of alcohols. Thus, the phenolic hydroxyl groups of tannic acid are able to react with the epoxy groups of GMA in the absence of catalyst. A hyperbranched methacrylates (TAHAs) was synthesized from tannic acid with GMA and glycidyl ester of versatic acid (CE10) (Figure 8) [58]. The content of methacrylate groups in the TAHAs was adjusted by controlling the ratio of GMA to CE10. The addition of TAHAs into AESO remarkably improved the hardness, adhesion, and tensile strength of the resulting coatings. 

### 3.3. Others

**Figure 9:**
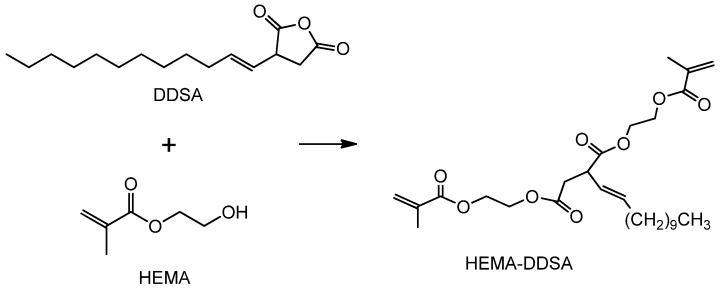
Synthesis of HEMA-DDSA from HEMA and DDSA [59].

Shen et al. [59] synthesized a multifunctional methacrylate (HEMA-DDSA) monomer from HEMA and 2-dodecen-1-ylsuccinic anhydride (DDSA) via esterification (Figure 9). The resulting monomer had relatively high molecular weight so that the volatility is insignificant. TMPTA has superior reactivity than styrene for its three unsaturated sites. Combination of HEMA-DDSA with TMPTA as co-crosslinker for UV-cured resins would accelerate the curing process [77].

**Figure 10:**
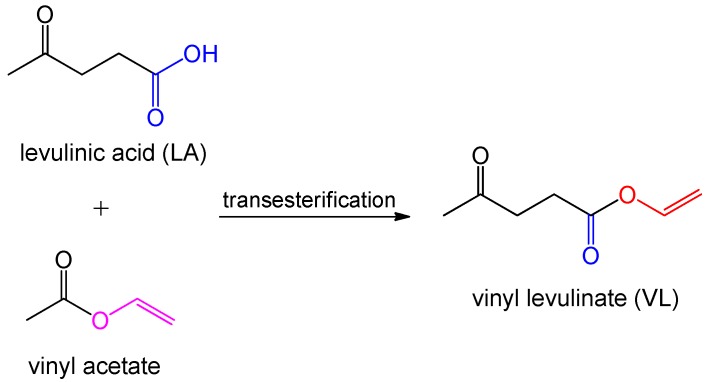
Synthesis of VL from levulinic acid and vinyl acetate [60].

Levulinic acid (LA) could be synthesized from hexose, which is hydrolyzed from biomass such as starch or cellulose. Levulinic acid contains a carboxylic group and a keto group, which could react with vinyl acetate via transesterification to form vinyl levulinate (VL) for use as a RD in UPE resins (Figure 10). However, the replacement of styrene with VL is limited due to its low reactivity and residual components that plasticize resin, hence weakening the crosslinking density and mechanical properties of the resulting resins [60].

**Figure 11:**
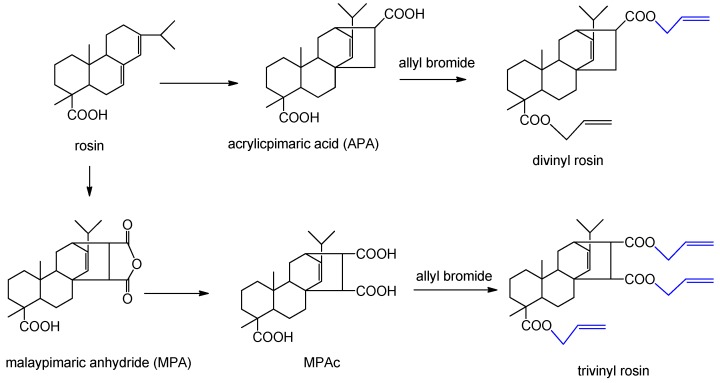
Synthesis of divinyl rosin and trivinyl rosin from rosin acid [61].

Rosin acid with rigid hydrogenated phenanthrene ring is beneficial for improving strength and modulus of the resulting resins [78]. Two kinds of rosin acid derivatives, i.e., malaypimaric anhydride and acrylicpimaric acid, were used to react with allyl bromide to obtain novel comonomers with divinyland trivinyl functionalities, respectively (Figure 11) [61]. The novel monomers were used as RDs for AESO resin, but their application is limited by their low reactivity. By contrast, a preferable curing system would be achieved by the reactions between rosin acid and GMA [78].

A series of biorenewable RDs from vanillin and eugenol were synthesized as possible styrene replacements in biobased VE thermosets [79]. In their routes, malonic acid was introduced into vanillin through piperidine-mediated addition, then the intermedia was obtained by an in situ double decarboxylation. After further methylation, the target compound 4-vinylveratrole was synthesized. In another route, vanillin was firstly allylated in solvents followed by aromatic-Claisen rearrangement. Similarly, the intermedia after rearrangement went through piperidine-mediated addition, decarboxylation and methylation to prepare 3-allyl-5-vinylveratrole. Following similar procedures, 3,5-diallyl-1,2-dimethoxybenzene was synthesized from eugenol. 

## 4. Properties of Styrene-Free Thermosets and Their Composites

### 4.1. Processibility of Thermosets

Processability should be primarily considered for the development of new resins in composites preparation. Viscosity of resin is one of the most important flow parameters to determine the end application of thermosetting resin. Viscosity not only affects the production cost of resin, but also is related to the properties and application of composites. Thus, in the process of developing resins with new RDs, the following factors that affect the viscosity of resin should be concerned. (1) Molecular weight of RDs: the increase of molecular weight can cause a sharp increase of apparent viscosity. Viscosity is directly related to the average molecular weight of resin. (2) Intermolecular force: generally, if there is an internal force such as hydrogen bond, the mobility of resin will be restricted and thus the viscosity increased [80]. For instance, the replacement of styrene with triglyceride in UPE significantly increased the viscosity of the resin system, which dramatically increased the difficulty of fabricating bamboo fibers reinforced composites [81].

Curing temperature of resins is closely related to the production cost of composites because of the required energy consumption. The maximum curing temperature of a resin system is determined by the reactivity of C=C bonds in the comonomers and the types of initiators. For the resins with different RDs, the curing temperature is affected by the reactivity of RDs and their reactivity ratio with main component. For example, the reactivity ratios (*r*) of VL-UPE (*r*_VL_ = 0.01 and *r*_UPE_ = 0.81) system are lower than 1 [60], which attests that both monomers are more likely to copolymerize rather than homopolymerize with a tendency toward alternation. To demonstrate the influence of RDs on the curing temperatures of resins, the AESO resin systems with different RDs initiated by *tert*-butyl perbenzoate (TBPB) were investigated. It is reported that NVP-AESO resin has higher curing temperature than styrene-AESO resin because of the high reactivity of NVP [30,82]. Similarly, isocyanatoethyl methacrylate (IEM) containing methacrylate groups has a much higher reactivity than styrene, which makes the maximum curing temperature of IEM-AESO resin significantly lower than that of styrene-AESO resin [83].

### 4.2. Properties of Thermosets and Their Composites

The dynamic and static mechanical properties of resins are contributed by their molecular structure and crosslinking degree, which further determines the properties of the composites when same fiber reinforcements were used. The reason why styrene is commonly used as RD is that its rigid benzene structure of styrene would endow rigidness on the resulting resins. For example, two bifunctional isocyanates, i.e., IEM and 3-isopropenyldimethylbenzyl isocyanate (TMI), were used as RDs for hemp fibers reinforced AESO composites, respectively [83]. Both IEM and TMI carries one C=C bond and one isocyanate group, which could concurrently improve the crosslinking density of AESO resins and the fiber-matrix interfacial adhesion of composites. However, the TMI-AESO composites presented much higher *T*_g_, storage modulus, tensile strength, tensile modulus, flexural strength and flexural modulus than the IEM-AESO composites because TMI monomer contains a benzene ring [83]. Thus, the renewable monomers with rigid structure such as lignin derivatives have been generally studied to develop biobased RDs. The VE resins mixed with MG or ME had comparable *T*_g_ with styrene-VE resins [53]. By contrast, the thermosets using fatty acids-derived RDs showed much lower *T*_g_ than others due to the flexible fatty acid chains [74]. 

On the other hand, crosslinking density reflects the curing degree and polymerization efficiency to some extent. For example, NVP with high reactivity would accelerate the free radical polymerization, resulting in the generation of cured resins with high crosslinking density [81]. BDDMA and TMPTMA were used to replace styrene in AESO system because they have more double bonds than styrene and hence can provide more unsaturated sites, resulting in resins with high crosslinking density and *T*_g_ [39]. According to the rubbery elastic theory, the calculated crosslinking density of IG-AESO and TMPTMA-AESO resins are 22921 and 46537 mol/m^3^, respectively, which are much higher than that of pure AESO (5612 mol/m^3^), leading to the higher *T*_g_ and modulus [72]. This proves that the *T*_g_ and stiffness of the crosslinked polymers have a close relationship with their crosslink density, which is in agreement with other polymer resin systems [84,85,86,87].

## 5. Conclusions

Many petroleum-based monomers could be directly used as styrene replacements and easily commercialized in thermosets and their composites. However, this is along with many drawbacks that limits their application. The synthesis of RDs from renewable resources is an increasingly interesting field. Several biobased RDs are available to replace petroleum feedstocks and highly volatile styrene. Their advantages are as follows: (1) The raw materials are renewable, reducing the use of fossil fuel resources; (2) new polymers with improved properties are produced; (3) they can replace styrene and reduce the emission of VOC. However, more systematic efforts are needed to realize commercial value that will enable them more competitive. Lignin derivatives show great prospects because they have similar structure to styrene. After the introduction of vinyl groups, they are believed to be the best alternative to styrene. In recent years, although some progress has been made in the production of biobased RDs with considerable properties, this field is still not fully developed. It is needed to do more work to promote the commercialization of these biobased RDs to the next stage.

## Figures and Tables

**Table 1:** Characteristics of commonly used vinyl monomers as reactive diluents (RDs).

Monomers	Molecular Weight (g/cm^3^)	Boiling Point (°C)	Toxicity	Chemical Structure
Styrene	104.15	145–146	acute toxicity	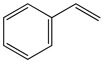
Divinylbenzene (DVB)	130.19	195	acute toxicity	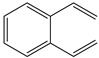
*α*-Methylstyrene	118.18	165–169	acute toxicity	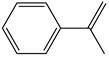
Fluorostyrene	122.14	67	-	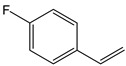
Vinyltoluene	118.18	169–171	acute toxicity	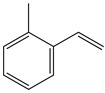
*N*-Vinyl-2-pyrrolidone (NVP)	111.14	92–95	acute toxicity	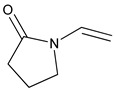
*tri*(Ethylene-glycol)divinyl ether (TDE)	202.25	120–126	-	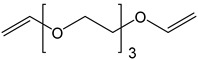

Note: The data were obtained from the data sheet provided by Sigma-Aldrich (St. Louis, MO, USA).

**Table 2:** Characteristics of commonly used (methyl)acrylate monomers.

Monomer	Molecular Weight (g/cm^3^)	Boiling Point (°C)	Toxicity	Chemical Structure
Butyl methacrylate (BMA)	140.20	162–165	acute toxicity	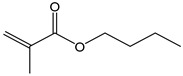
Hydroxypropylacrylate (HPA)	130.14	77	acute toxicity	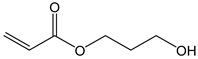
2-Hydroxyethylmethac-rylate (HEMA)	130.14	85	danger	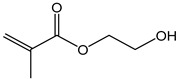
Isobornyl methacrylate (IBOMA)	222.32	127–129	environmental hazards	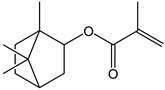
Methyl methacrylate (MMA)	100.12	100	acute toxicity	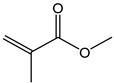
Trimethylolpro-panetri-acrylate (TMPTA)	296.32	-	acute toxicity	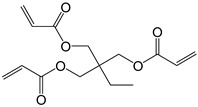
Trimethylolpropane trimethacrylate (TMPTMA)	338.40	-	environmental hazards	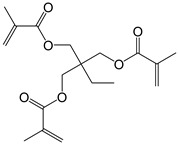
1,4-Butanediol dimethacrylate (BDDMA)	226.27	132–134	acute toxicity	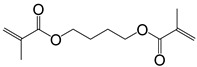

Note: The data were obtained from the data sheet provided by Sigma-Aldrich (St. Louis, MO, USA).

**Table 3:** Synthesis of novel monomers from renewable materials.

Methods	Renewable Monomers	Reactive Agents	Novel Monomer	Sources of Monomer
Grafting acrylate	cardanol	acryloyl chloride (AC)	cardanyl acrylate (CA) [44]	cashew nut
	castor oil	AC	a new polyfunctional acrylate monomer (COPERAA) [45]	castor
	betulin	AC	acrylated betulin [46,47]	rosin
Grafting methacr-ylate	cardanol	methacrylic acid	methacrylated cardanol (MC) [48,49]	cashew nut
	cardanol	methacrylic anhydride (MA)	methacrylated cardanol (MC) [50]	cashew nut
	sobrerol	MA	sobrerol methacrylate (SoMet) [51]	α-pinene
	isosorbide	MA	methacrylated isosorbide (MI) [52]	starch/cellulose
	vanillin	MA	methacrylated vanillin (MV) [53]	lignin
	guaiacol	MA	methacrylated guaiacol (MG) [53]	lignin
	eugenol	MA	methacrylated eugenol (ME) [53]	lignin
	phenol	MA	phenyl methacrylate (PM) [54]	lignin
	creosol	MA	methacrylated creosol (MCre) [54]	lignin
	4-ethylguai-acol	MA	methacrylated 4-ethylguaiacol (MEG) [54]	lignin
	4-propylgu-aiacol	MA	methacrylated 4-propylguaiacol (MPG) [54]	lignin
	catechol	MA	methacrylated catechol (MCat) [54]	lignin
	4-methylcat-echol	MA	methacrylated 4-methylcatechol (MMCat) [54]	lignin
	vanillin alcohol	MA	methacrylated vanillyl alcohol (MVA) [55]	lignin
	furoic acid	glycidyl methacrylate (GMA)	furoic acid glycidyl methacrylate (FA-GM) [56]	cellulose
	itaconic acid	GMA	a UV curable unsaturated monomer (IG) [57]	starch/cellulose
	oleic acid	Br_2_/GMA	9-10 dibromo stearic acid glycidyl methacrylate [3]	extractives
	oleic acid	GMA	methacrylated fatty acid (MFA)	extractives
	tannic acid	GMA/glycidyl ester of versatic acid (CE10)	hyperbranched methacrylates [58]	starch/cellulose
Others	Hydroxyeth-ylmethacry-late (HEMA)	2-dodecen-1-ylsuccinic anhydride	a dimethacrylate reactive diluent (HEMA-DDSA) [59]	-
	levulinic acid (LA)	vinyl acetate	vinyl levulinate (VL) [60]	starch/cellulose
	rosin derivatives	allyl bromide	divinyl rosin/trivinyl rosin [61]	rosin

## Abbreviations

AC, acryloyl chloride; AESO, acrylated epoxidized soybean oil; BDDMA, 1,4-butanediol dimethacrylate; BMA, butyl methacrylate; CA, cardanyl acrylate; COPERAA, a new polyfunctional acrylate monomer; DI, dimethyl itaconate; DVB, divinyl benzene; FA-GM, furoic acid glycidyl methacrylate; HEMA, hydroxyethyl methacrylate; HEMA-DDSA, a dimethacrylate reactive diluent; HPA, hydroxypropyl acrylate; IBOMA, isobornyl methacrylate; IEM, isocyanatoethyl methacrylate; IG, a UV curable unsaturated monomer; LA, levulinic acid; MAA, methyl methacrylate; MACO, maleated castor oil; MC, methacrylated cardanol; MCat, methacrylated catechol; MCre, methacrylated creosol; ME, methacrylated eugenol; MEG, methacrylated 4-ethylguaiacol; MFA, methacrylated fatty acid; MG, methacrylated guaiacol; MMCat, methacrylated 4-methylcatechol; MI, Methacrylated isosorbide; MPG, methacrylated 4-propylguaiacol; MV, methacrylated vanillin; MVA, methacrylated vanillyl Alcohol; NVP, *N*-vinyl-2-pyrrolidone; PM, phenyl methacrylate; RDs, Reactive diluents; SoMet, sobrerol methacrylate; TDE, *tri*(ethylene glycol) divinyl ether; TMI, 3-isopropenyldimethylbenzyl isocyanate; TMPTA, trimethylolpropane triacrylate; TMPTMA, trimethylolpropane trimethacrylate; UPE, unsaturated polyester; VE, vinyl ester; VL, vinyl levulinate; VOC, volatile organic compound.
